# Dietary Intake of *Trans* Fatty Acids in Children Aged 4–5 in Spain: The INMA Cohort Study

**DOI:** 10.3390/nu8100625

**Published:** 2016-10-10

**Authors:** Alexander Scholz, Daniel Gimenez-Monzo, Eva Maria Navarrete-Muñoz, Manuela Garcia-de-la-Hera, Ana Fernandez-Somoano, Adonina Tardon, Loreto Santa Marina, Amaia Irazabal, Dora Romaguera, Mònica Guxens, Jordi Julvez, Sabrina Llop, Maria-Jose Lopez-Espinosa, Jesus Vioque

**Affiliations:** 1 Unit of Nutritional Epidemiology, Universidad Miguel Hernandez, Alicante 03550, Spain; scholz.alexander@gmail.com (A.S.); dgimenez@umh.es (D.G.-M.); enavarrete@umh.es (E.M.N.-M.); manoli@umh.es (M.G.-d.-l.-H.); 2 CIBER de Epidemiología y Salud Pública (CIBERESP), Madrid 28029, Spain; capua.uo@uniovi.es (A.F.-S.); atardon@uniovi.es (A.T.); ambien4ss-san@euskadi.eus (L.S.M.); mguxens@isglobal.org (M.G.); jjulvez@isglobal.org (J.J.); llop_sab@gva.es (S.L.); lopez_josesp@gva.es (M.-J.L.-E.); 3 Department of Medicine, University of Oviedo, Oviedo 33006, Spain; 4 BIODONOSTIA, Instituto de Investigación Biosanitaria, San Sebastián 20014, Spain; amaia-irazabal@hotmail.es; 5 Subdirección Salud Publica Gipuzkoa, San Sebastián 20013, Spain; 6 Instituto de Investigacion Sanitaria de Palma (IdISPa), Hospital Universitario Son Espases, Palma de Mallorca 07120, Spain; d.romaguera-bosch@imperial.ac.uk; 7 CIBER Fisiopatología de la Obesidad y Nutrición (CIBEROBN), Madrid 28029, Spain; 8 ISGlobal, Centre for Research in Environmental Epidemiology (CREAL), Barcelona 08036, Spain; 9 Pompeu Fabra University, Barcelona 08003, Spain; 10 Department of Child and Adolescent Psychiatry/Psychology, Erasmus University Medical Centre-Sophia Children’s Hospital, Rotterdam 3000CD, The Netherlands; 11 Epidemiology and Environmental Health Joint Research Unit, FISABIO–Universitat Jaume I–Universitat de València, Valencia 46020, Spain

**Keywords:** *trans* fatty acids, dietary fats, risk factors, preschool child

## Abstract

*Trans* fatty acid (TFA) intake has been identified as a health hazard in adults, but data on preschool children are scarce. We analyzed the data from the Spanish INMA Project to determine the intake of total, industrial and natural TFA, their main sources and the associated socio-demographic and lifestyle factors in children aged 4–5 (*n* = 1793). TFA intake was estimated using a validated Food Frequency Questionnaire, and multiple linear regression was used to explore associated factors. The mean daily intakes of total, industrial and natural TFA were 1.36, 0.60, and 0.71 g/day, respectively. Ten percent of the children obtained >1% of their energy intake from TFA. The main sources of industrial TFA were fast food, white bread and processed baked goods. Milk, red and processed meat and processed baked goods were the main sources of natural TFA. Having parents from countries other than Spain was significantly associated with higher natural TFA (in mg/day) intake (β 45.5) and television viewing was significantly associated with higher industrial TFA intake (β 18.3). Higher fruits and vegetables intake was significantly associated with lower intakes of all TFAs, whereas higher sweetened beverages intake was significantly associated with lower total and natural TFA intake. Thus, total and industrial TFA intake was associated with less healthy food patterns and lifestyles in Spanish preschool children.

## 1. Introduction

*Trans* fatty acids (TFA) are geometrical isomers of unsaturated fatty acids and have at least one non-conjugated carbon-carbon double bond in the *trans* configuration [1]. Dietary TFA can be of natural (ruminant) or artificial origin. The artificial TFA are industrially produced by partial hydrogenation of unsaturated vegetable oils [2]. This process occurs when vegetable oils are heated and it is industrially used to harden vegetable oils into margarine and shortening. It also occurs during deep fat frying. The advantages of the industrial use of hardened vegetable oils include low costs and longer shelf lives. The main sources of industrial TFA are margarines, commercially baked products, deep-fried fast foods, packaged snack foods and other prepared foods [3]. On the other hand, natural TFA are ingested by the consumption of meat or dairy products from ruminant animals where small amounts of TFA are produced by microorganisms present in the rumen [2].

In adults, high TFA intake has been associated with an increased cardiovascular risk, mainly due to low density lipoprotein cholesterol increase, high-density lipoprotein cholesterol decrease, pro-inflammatory effects and the promotion of endothelial dysfunction [3,4]. Furthermore, high TFA intake may worsen insulin sensitivity [3,4]. However, the evidence for the relation between TFA intake and diabetes is not consistent [3]. Additionally, high TFA has been identified as a risk factor for weight gain or higher weight [4,5]. A recently published meta-analysis has confirmed that TFA intake is associated with all-cause mortality and coronary heart disease mortality [6]. The negative health effects of TFA have been attributed mainly to TFA in general or TFA of industrial origin and some studies have suggested that natural TFA might have beneficial properties [7]. Nevertheless, the role of the different TFA subtypes is not fully clear and further research is needed in order to understand whether the negative effects of TFA consumption depends on their industrial or natural origin, or both [8]. Because of the demonstrated health hazards, many countries have established legal limits regarding the content of TFA in foods and the World Health Organization recommends a consumption of less than 1% of energy from TFA [9].

A recent Canadian study of children aged 5–6 reported that the mean total TFA intake was 0.71 energy % and that 12% of the participants consumed >1% of the energy from TFA [10]; the authors concluded that more efforts should be undertaken to enable the selection of foods which are lower in TFA in order to further reduce the TFA intake in children. However, the published information about the sources and amounts of TFA intake for preschool children is scarce and, to our knowledge, no study has distinguished yet between industrial and natural TFA intake in this population [11,12].

Therefore, the objective of this study was to estimate the intake and major sources of total, industrial, and natural TFA as well as to identify the factors associated with a high TFA intake for children aged 4–5 from a Spanish multicenter prospective mother-child cohort study, the INMA-Project [13].

## 2. Materials and Methods

### 2.1. Study Design and Participants

The INMA project (*INfancia y Medio Ambiente*, Spanish for “Childhood and Environment”) is a Spanish multicenter prospective mother-child cohort study which consists of a network of birth cohorts with a common study protocol in the regions of Asturias, Guipuzcoa, Sabadell and Valencia. It aims to study the effect of diet and environmental factors during pregnancy and early childhood on child growth, development and health. The detailed study protocol was published elsewhere [13]. Briefly, between November 2003 and August 2008 pregnant women were recruited and 2644 women agreed to participate. Finally, 2506 mothers and their newborns met the inclusion criteria and were included in the study. Of these children 1829 (73.0%) were evaluated at the age of 4–5 years between 2009 and 2012. For the present analysis, we excluded the participants with missing data for the variables of interest. Finally, 1793 children (98.0%) were included (387 from Asturias, 395 from Guipuzcoa, 429 from Sabadell and 582 from Valencia) and the data at the age of 4–5 was cross-sectionally analyzed. The study was approved by the Ethical Committee of the Municipal Institute of Medical Investigation (DSP-JV 001-11) and by the Ethical Committees of the centers involved in the study (Hospital La Fe, Valencia; Hospital de Zumarraga, Guipuzcoa; Hospital de Sabadell, Sabadell; Hospital Universitario Central de Oviedo, Asturias, Spain). The pregnant women were informed about the study and informed consent was obtained at each of the visits.

### 2.2. Dietary Assessment

We used a semi-quantitative food frequency questionnaire (FFQ) of 105 food items to assess the usual diet (available at: http://bibliodieta.umh.es/files/2011/07/CFA105.pdf). The FFQ was derived from an adult version of the FFQ previously validated among the mothers of the INMA Study [14]. The FFQ was modified to include foods and portion sizes appropriate to children aged 4–5 and further validated with three 24-h recalls and several biomarkers in a sample of 169 children aged 4–5 from the Valencia cohort [15]. To address the reproducibility and validity against the 24-h recalls Pearson’s correlations adjusted for energy intakes were performed and the correlation coefficients were 0.44 and 0.23, respectively; in both cases statistically significant [15]. The range of the reproducibility and validity coefficients of the FFQ was similar to those observed for other FFQs in the literature [16].

Parents were asked to report how often, on average, their children had consumed the specified serving or portion size for each food item of the FFQ in the previous year. The questionnaire had nine possible responses, ranging from “never or less than once per month” to “six or more per day”.

Nutrient values were primarily obtained from food composition tables from the US Department of Agriculture [17], other published sources reporting information on TFA content in Spanish foods and other sources with detailed information for total, industrial and natural TFA in foods [18,19,20,21,22]. We calculated the usual daily nutrient intakes for each child by multiplying the frequency of the use of each food item by the nutrient content of the portion size specified in the FFQ. Then we added all foods to obtain the total nutrient intake for each participant. The usual daily intake of TFA was expressed in g/day and and for the linear regression analysis it was converted into mg/day. Furthermore, we estimated the energy intake in kcals/day for each participant. We used the residual method to estimate calorie-adjusted values for the nutrient intakes [23]. The fruit and vegetable intake as well as the sweetened beverage intake were estimated and expressed in 100 g/day.

To estimate the main food sources of total, industrial and natural TFA intakes, we grouped the consumed foods as follows: White bread, fast food (French fries, pizza, pies, chips and other salty snacks), processed baked goods (including cakes and cupcakes, Maria biscuits, chocolate cookies, buns, croissants and donuts), sweets (including chocolates, hazelnut spread and instant chocolate drinks), ice cream, margarine, red and processed meat (including beef, pork, lamb, sausages and hamburger meat), milks (including whole milk, fat reduced milk, energy milk and milkshakes), yoghurts (including natural, sugared or with fruits and cheese yoghurts), cheese (hard cheese and cream cheese), butter and others. The contributions of each food or food group to the total, industrial and natural TFA intake were added and the results were presented graphically.

### 2.3. Covariates

The following parental information was collected at baseline in pregnancy: Maternal age (years), paternal age (years), country of origin (both parents from Spain, otherwise), mother’s educational level (none or primary, secondary, university), mother’s social class (based on the Spanish adaptation of the Register General’s Social Class classification: low, IV-V; middle, III; high, I-II). Additionally, the following child characteristics were collected: sex (male, female), age (in years), parentally self-reported physical activity (sedentary, active) and TV viewing (in hours/day). The usual daily intake of fruit and vegetables (in units of 100 g/day) and sweetened beverages (in units of 100 g/day) among children at the age of 4–5 years were estimated from the FFQ administered to the parents.

### 2.4. Statistical Analysis

We performed descriptive analyses to compare both, the TFA intake in g/day and energy-percent, according to parental and children’s characteristics and cohort. We applied one-way analysis of variance (ANOVA) for continuous variables and Chi-squared tests for categorical variables. We used multiple linear regression to estimate adjusted β regression coefficients and 95% confidence intervals in order to explore the association between parental and children’s characteristics and the intake of total, industrial, and natural TFA (in mg/day). To explore the factors associated with TFA intake and to control for possible confounding variables, all multivariable models were adjusted for maternal age, paternal age, country of origin, mother’s educational level, mother’s social class, child’s sex, child’s age, parentally reported physical activity of the child, TV viewing, fruit and vegetable intake, sweetened beverage intake and mean daily energy intake. We used meta-analysis to obtain combined estimates from cohort specific linear regression models and the I^2^ measure under the fixed effects hypothesis to explore heterogeneity among cohorts [24]. We applied the fixed-effects meta-analysis model when *I*^2^ was <50% and the random-effects meta-analysis model when *I*^2^ ≥ 50%. All the statistical analyses were conducted with the statistical software R, version 3.2.3 (R Foundation for Statistical Computing, Vienna, Austria). All statistical tests were bilateral and *p*-values < 0.05 were considered statistically significant.

## 3. Results

Table 1 shows detailed information about the intake of total, industrial and natural TFA. The mean daily intake for total TFA was 1.36 g/day (median 1.30 g/day) and the corresponding intakes for industrial and natural TFA were 0.60 and 0.71 g/day respectively. Thus, slightly more than half of the total TFA intake came from natural TFA. Significant differences were observed in mean daily intakes of TFA by cohort with Valencia showing the highest total and industrial TFA intake and Guipuzcoa the lowest intakes for all types of TFA (*p* < 0.05). Regarding the percentage of the total energy intake provided by TFA, 10% of the children obtained at least 1% of their energy intake from TFA which approximately corresponded to an absolute mean daily intake of 1.95 g/day; this percentage ranged from 6.5% in children from Sabadell to 14.6% in children from Valencia. Statistically significant differences were also observed for several parental and child characteristics among cohorts (see Supplementary Table S1).

**Figure 1:**
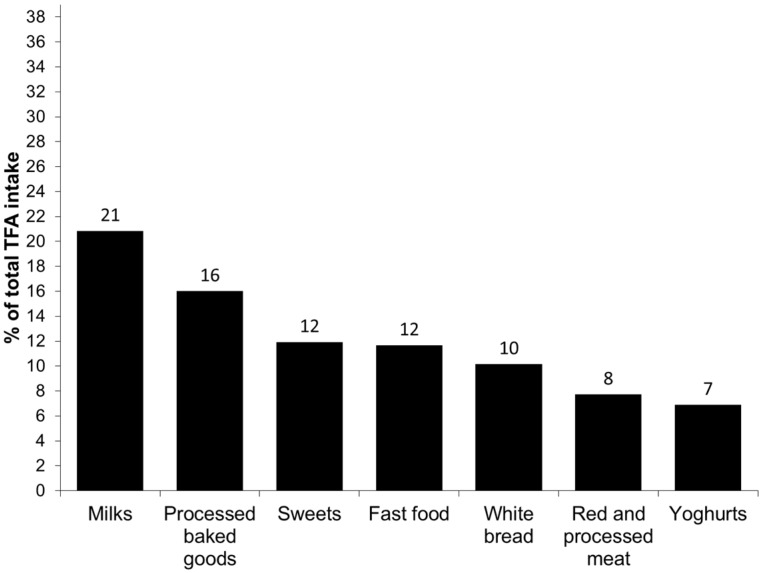
Main food sources of the intake of total *trans* fatty acids (TFA) in children aged 4–5 from the INMA Study.

**Figure 2:**
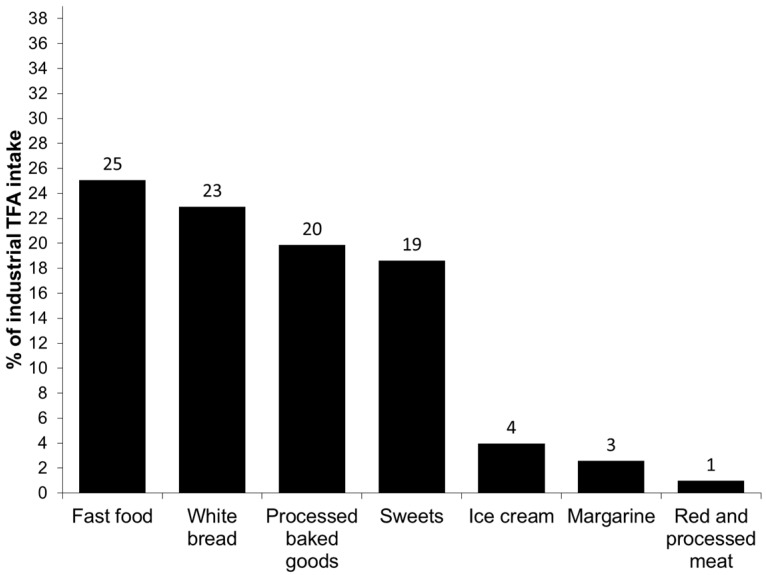
Main food sources of the intake of industrial *trans* fatty acids (TFA) in children aged 4–5 from the INMA Study.

**Figure 3:**
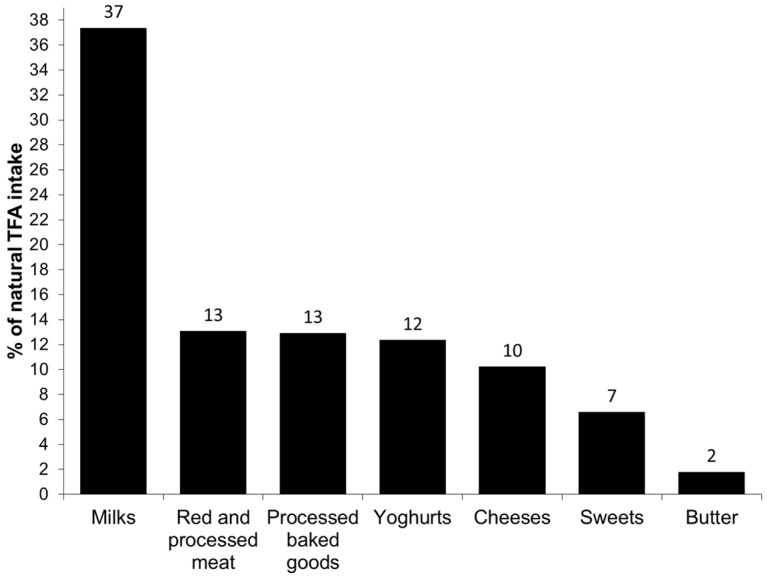
Main food sources of the intake of natural *trans* fatty acids (TFA) in children aged 4–5 from the INMA Study.

The principal food groups contributing to the total, industrial and natural TFA intake are presented in Figure 1, Figure 2 and Figure 3, respectively. The major source of total TFA intake in children were milks (21%), processed baked goods (16%), sweets (12%), fast food (12%), white bread (10%), red and processed meat (8%) and yoghurts (7%). The major contributors to the industrial TFA intake were white bread (25%), fast food (23%), processed baked goods (20%) and sweets (19%), approximately accounting for 87% of the intake. The most important contributor to the intake of natural TFA were milks (37%), followed by red and processed meat (13%), processed baked goods (13%), yoghurts (12%) and cheeses (10%).

Table 2 shows the association between parental and child characteristics and the intakes of total, industrial and natural TFA (in mg/day). Having parents not originally from Spain was associated with a higher intake of total TFA (β 45.5, 95% CI 1.3; 89.6) and natural TFA (β 65.7, 95% CI 37.4; 94.0). TV viewing (hours/day) was associated with a higher industrial TFA intake (β 18.3, 95% CI 5.9; 30.7). The intake of fruits and vegetables (100 g/day) was inversely associated with the intakes of total, industrial and natural TFA (β −85.5, β −46.6, and β −36.9, respectively; *p* < 0.05). A higher intake of sweetened beverages (100 g/day) was associated with a lower total (β −27.6) and natural (β −34.8) TFA intake (*p* < 0.05).

## 4. Discussion

To our knowledge this is the first study that reports the intake of TFA in preschool children aged 4–5 years distinguishing industrial and natural TFA. We show that in this population more than 50% of the total TFA intake was of natural origin and that about 10% of the children of the INMA study obtained at least 1% of their energy intake from TFA, thus exceeding the limit recommended by the WHO [9].

Our results are in part comparable to those reported by a Canadian study with 100 children aged 5 to 6 [10], although this study did not report the absolute TFA intake. The mean percentage of energy intake provided by TFA was 0.71% in the Canadian study, and therefore similar than in our study (0.77%). The percentage of children exceeding 1% of the energy intake from TFA was also comparable to that in our study (12% vs. 10%). In a survey conducted in a Chinese population with participants aged 3 years and older, the reported intakes of total TFA among children from 3 to 6 years were much lower than those in our study; the mean daily TFA intake was 0.49 g/day which corresponded to 0.34% of their total energy intake, and less than 0.42% of the participants surpassed the limits recommended by the WHO [25].

In our study population 52% of the ingested TFA were of natural origin. The average intake of industrial TFA reported in most studies in adult populations has been about 2.5-fold higher than that of natural TFA [6]. The higher percentage of the intake of natural TFA reflects the differences between adults and children regarding their nutrition and may be due to a relative higher intake of dairy products. Moreover, this makes preschool children of special interest to study the effects of natural TFA on health outcomes. There is evidence that industrial TFA, but not natural TFA, are associated with a higher risk of coronary heart disease [6]. This could be explained by a true effect of the ingestion of industrial TFA, but also be the result of lower consumption levels of natural TFA in adults. Therefore, it has been suggested that more research is required to assess the impact of natural versus industrially produced TFA on health outcomes. Thus, preschool children may be an interesting study population because of the proportion of their natural and industrial TFA intakes.

The main food sources of the total TFA intake in our study were comparable to those reported by the Canadian study [10]. Regarding the industrial TFA intake, we observed that the main sources were fast food, white bread, processed baked goods and sweets, contributing to nearly 90% to its intake. The important contribution of white bread to the industrial TFA intake is interesting and probably due to high white bread consumption in the Spanish study population, but also underlines the importance of bread and bakery products as TFA sources. Additionally, the main sources of industrial TFA obtained in the present study confirm that industrial TFA intake marks a highly processed diet which might contribute to negative health effects. On the other hand, the principal contributors to the natural TFA intake were milk and dairy products, foods highly important in the diet of preschool children.

Having parents from a country of origin other than Spain was significantly associated with a higher total TFA intake, which was based on a substantially higher natural TFA intake as no significant difference was observed for industrial TFA. The parents who were not from Spain came from many different European and non-European countries and the observed differences could be due to different dietary habits. Interestingly, more TV viewing was associated with a higher industrial, but not natural TFA intake. This finding can be explained by the well-established association between TV viewing and the consumption of fast food or poor diet in general [26,27,28]. Moreover, a higher intake of fruit and vegetables was associated with a lower intake of total, industrial and natural TFA which indicates that the consumption of industrial and natural TFA containing foods was probably replaced by fruits and vegetables. This association could be similar to the inverse association observed between fruit and vegetable intake and fat intake reported by several studies [29,30]. The inverse association between the intake of sweetened beverages and the total TFA intake was based on a lower intake of natural TFA, as no significant difference was observed with industrial TFA. The lower natural TFA intake is probably based on the replacement of milk by sweetened beverages in the diet of children with high sweetened beverage ingestion, which was also shown in a study carried out among three to seven-year-old children [31].

A limitation of this study may be that the estimates of TFA intakes were based on the children’s food intakes reported by their parents using a FFQ. The reproducibility and validity of the FFQ was assessed for many foods and nutrients, including total TFA intake, against three 24-h recall but not for industrial and natural TFA intakes. However, we were not able to validate the biochemical validity of the FFQ against plasma concentration of total, industrial or natural TFA although it may not be adequate to use TFA in plasma as the reference method since there is evidence that TFA may selectively accumulate in some tissues; adipose tissue and liver generally contain higher concentrations of TFA than blood or other tissues, which could explain the lack of or very low correlations between TFA plasma concentration and the TFA intake reported by some studies [32,33]. Furthermore, an overestimation of total and industrial TFA intakes may have occurred in our study since the industrial TFA content in some foods (e.g., margarine) may have decreased during the last few years in Spain [19]. However, the food composition table primarily used was revised in 2015 [17], but some additional information was published between 2003 and 2011 [19,20,21,22], which may have led to an overestimation if compared to the current industrial TFA content of commercial products. Nevertheless, the data in our study was obtained between 2009 and 2012 and the internal validity of our study should not be affected by the latest changes in TFA contents.

Despite some limitations, to our knowledge this study is the first assessment of the TFA intake in children in Spain that distinguishes between industrial and natural sources. Additionally, it provides helpful information on TFA intake and the main food sources, which may be helpful to modify present legislations about the allowed TFA content of foods. Moreover, the information provided could help to design dietary guidelines in order to reduce the ingestion of TFA, ideally by replacing them by other *cis* unsaturated fatty acids [34].

## 5. Conclusions

The present study shows that the TFA intake is too high in a relevant proportion of children in Spain. It should be reduced to prevent possible future health problems, such as cardiovascular diseases or obesity. In contrast to findings in adult populations, the intake of natural TFA is higher than the industrial TFA intake. Further research is needed to investigate the specific effects of natural and industrial TFA in the pathogenesis of diseases. Meanwhile, their exact roles remain unclear, parents should be aware of the main sources of TFA in order to reduce the total TFA intake of their children.

## Figures and Tables

**Table 1:** Intakes of total, industrial, and natural trans fatty acids (TFA) among children aged 4–5 years from the four cohorts of the INMA study.

	All Cohorts (*n* = 1793)		Asturias (*n* = 387)		Guipuzcoa (*n* = 395)		Sabadell (*n* = 429)		Valencia (*n* = 582)	
	g/day	E% ^1^	g/day	E% ^1^	g/day	E% ^1^	g/day	E% ^1^	g/day	E% ^1^
Total TFA intake										
Mean ^2^	1.36	0.77	1.40	0.76	1.22	0.74	1.35	0.74	1.43	0.81
S.D. ^2^	0.45	0.17	0.47	0.16	0.38	0.17	0.41	0.17	0.50	0.18
Percentile ^3^										
P10	0.85	0.56	0.89	0.56	0.77	0.54	0.89	0.56	0.86	0.59
P25	1.03	0.65	1.08	0.65	0.93	0.62	1.07	0.63	1.06	0.68
P50	1.30	0.75	1.32	0.74	1.17	0.74	1.29	0.73	1.38	0.80
P75	1.60	0.87	1.59	0.86	1.46	0.85	1.60	0.83	1.73	0.92
P90	1.95	1.00	1.97	0.99	1.67	0.98	1.90	0.94	2.11	1.05
Industrial TFA intake										
Mean ^2^	0.60	0.34	0.54	0.30	0.54	0.33	0.63	0.35	0.65	0.37
S.D. ^2^	0.27	0.13	0.26	0.11	0.21	0.11	0.27	0.13	0.30	0.13
Percentile ^3^										
P10	0.31	0.20	0.28	0.17	0.31	0.21	0.35	0.21	0.33	0.21
P25	0.42	0.26	0.38	0.22	0.39	0.25	0.45	0.26	0.45	0.28
P50	0.55	0.32	0.49	0.29	0.50	0.32	0.57	0.33	0.59	0.35
P75	0.73	0.40	0.64	0.35	0.66	0.38	0.76	0.41	0.81	0.44
P90	0.94	0.50	0.85	0.43	0.81	0.46	0.98	0.52	1.06	0.55
Natural TFA intake										
Mean ^2^	0.71	0.40	0.82	0.45	0.64	0.39	0.65	0.36	0.72	0.41
S.D. ^2^	0.28	0.12	0.31	0.13	0.26	0.12	0.23	0.10	0.28	0.11
Percentile ^3^										
P10	0.40	0.26	0.48	0.29	0.36	0.23	0.40	0.23	0.41	0.27
P25	0.51	0.32	0.59	0.35	0.46	0.30	0.49	0.29	0.52	0.34
P50	0.67	0.39	0.80	0.43	0.60	0.38	0.62	0.35	0.70	0.40
P75	0.86	0.47	0.97	0.53	0.76	0.46	0.79	0.43	0.88	0.48
P90	1.10	0.56	1.21	0.62	0.97	0.55	0.95	0.49	1.11	0.56

^1^ E%, energy percent (percentage of the total ingested energy); ^2^ Mean and standard deviation of the daily TFA intake in g/day and as percentage of the total energy intake provided by TFA intake. The tests of analysis of variance were significant in all comparisons (*p* < 0.05); ^3^ Percentiles of the TFA intake: P10, P25, P50, P75, P90.

**Table 2:** Associations between parental and child characteristics and the intake of total, industrial, and natural *trans* fatty acid (TFA) among the children aged 4–5 years of the INMA Study.

	Total TFA (mg/Day)			Industrial TFA (mg/Day)			Natural TFA (mg/Day)		
	β ^1^	95% CI	*I*^2^, % ^2^	β ^1^	95% CI	*I*^2^, % ^2^	β ^1^	95% CI	*I*^2^, % ^2^
*Parental characteristics*									
Maternal age, years	−1.6	−11.8; 8.6	81.3 ^3^	0.7	−4.4; 5.8	57.8	−2.2	−7.4; 2.9	64.7 ^3^
Paternal age, years	−0.6	−4.2; 3.1	59.6	−0.5	−3.2; 2.3	0.0	−0.3	−6.1; 5.5	81.0 ^3^
Country of origin other than Spain	45.5	1.3; 89.6	0.0	−13.6	−47.8; 20.5	0.0	65.7	37.4; 94.0	0.0
Mother’s educational level									
Primary or no education	ref.	ref.		ref.	ref.		ref.	ref.	
Secondary	−11.0	−48.1; 26.0	25.6	−1.5	−42.9; 40.0	53.0	−17.6	−42.1; 6.9	25.9
University	−8.0	−82.6; 66.6	61.3	15.3	−49.7; 19.2	35.2	13.9	−16.8; 44.6	44.0
Mother’s social class									
Low	ref	ref		ref	ref		ref	ref	
Middle	11.1	−28.2; 50.3	0.0	0.4	−28.1; 29.6	26.3	5.0	−21.9; 31.9	0.0
High	−1.1	−43.0; 40.8	31.2	−20.4	−51.5; 10.7	20.4	16.0	−12.7; 44.7	0.0
*Child’s characteristics*									
Physical activity (parentally reported)									
Sedentary	ref.	ref.		ref.	ref.		ref.	ref.	
Active	−1.0	−28.7; 26.6	0.0	−16.4	−37.0; 4.2	0.0	12.2	−6.5; 30.9	0.0
TV viewing, h/day	16.0	−0.5; 32.6	39.9	18.3	5.9; 30.7	0.0	−2.9	−13.9; 8.1	22.4
Fruit and vegetable intake, 100 g/day	−85.5	−104.8; −66.1	63.6 ^3^	−46.6	−61.9; −31.3	67.7 ^3^	−36.9	−44.9; −28.9	40.3
Sweetened beverage intake, 100 g/day	−27.6	−41.1; −14.0	0.0	8.5	−1.7; 18.7	0.0	−34.8	−43.6; −26.0	0.0

^1^ The cohort specific linear regression models were combined using meta-analysis. All models were adjusted for the child’s sex, age, energy intake and all variables in the table. ^2^ We used the fixed-effects meta-analysis model when *I*^2^ was <50% and the random-effects meta-analysis model when *I*^2^ ≥ 50%. ^3^ *p*_Cochran Q_ < 0.05 from the test of heterogeneity.

## Supplementary Materials

The following are available online at http://www.mdpi.com/2072-6643/8/10/625/s1, Table S1: Parental and child characteristics of the children aged 4–5 years in all cohorts of the INMA Study.
