# Supplementary material for: Dietary Intake of Trans Fatty Acids in Children Aged 4–5 in Spain: The INMA Cohort Study
Source: Nutrients. 2016 Oct 10;8(10):625. doi: 10.3390/nu8100625 (PMC5084013; doi:10.3390/nu8100625)
Supplement: Supplementary file 1 [file nutrients-08-00625-s001.docx]

Supplementary Materials: Dietary Intake of *Trans* Fatty Acids in Children Aged 4–5 in Spain: The INMA Cohort Study

Alexander Scholz, Daniel Gimenez-Monzo, Eva Maria Navarrete-Muñoz, Manuela Garcia de la Hera, Ana Fernandez-Somoano, Adonina Tardon, Loreto Santa Marina, Amaia Irazabal, Dora Romaguera, Monica Guxens, Jordi Sunyer, Sabrina Llop, Maria-Jose Lopez-Espinosa, Jesus Vioque

**Table S1.** Parental and child characteristics of the children aged 4–5 years in all cohorts of the INMA Study.

|  | **All Cohorts (*n* = 1793)** | | **Asturias (*n* = 387)** | | **Guipuzcoa (*n* = 395)** | | **Sabadell (*n* = 429)** | | **Valencia (*n* = 582)** | |  |
| --- | --- | --- | --- | --- | --- | --- | --- | --- | --- | --- | --- |
| **Variable** | **Mean or *n*** | **SD or %** | **Mean or *n*** | **SD or %** | **Mean or *n*** | **SD or %** | **Mean or *n*** | **SD or %** | **Mean or *n*** | **SD or %** | ***p*-Value ^a^** |
| *Parental characteristics* |  |  |  |  |  |  |  |  |  |  |  |
| Maternal age at pregnancy, years | 31.0 | 4.1 | 31.9 | 4.2 | 31.5 | 3.4 | 30.5 | 4.2 | 30.4 | 4.2 | <0.001 |
| Paternal age, years | 33.0 | 4.8 | 34.1 | 5.1 | 33.9 | 4.4 | 32.3 | 4.7 | 32.2 | 4.7 | <0.001 |
| Country of origin Spain | 1594 | 88.9 | 363 | 93.8 | 383 | 97.0 | 365 | 85.1 | 483 | 83.0 | <0.001 |
| Mother´s educational level |  |  |  |  |  |  |  |  |  |  | <0.001 |
| None or primary | 386 | 21.5 | 64 | 16.5 | 46 | 11.6 | 109 | 25.4 | 167 | 28.7 |  |
| Secondary | 741 | 41.3 | 166 | 42.9 | 139 | 35.2 | 180 | 42.0 | 256 | 44.0 |  |
| University | 666 | 37.1 | 157 | 40.6 | 210 | 53.2 | 140 | 32.6 | 159 | 27.3 |  |
| Mother´s social class |  |  |  |  |  |  |  |  |  |  | <0.001 |
| High (I-II) | 879 | 49.0 | 211 | 54.5 | 149 | 37.7 | 194 | 45.2 | 107 | 18.4 |  |
| Middle (III) | 483 | 26.9 | 82 | 21.2 | 112 | 28.4 | 139 | 32.4 | 150 | 25.8 |  |
| Low (IV-V) | 431 | 24.0 | 94 | 24.3 | 134 | 33.9 | 96 | 22.4 | 325 | 55.8 |  |
| *Child characteristics* |  |  |  |  |  |  |  |  |  |  |  |
| Child´s sex female | 862 | 48.1 | 180 | 46.5 | 200 | 50.6 | 207 | 48.3 | 275 | 47.3 | 0.664 |
| Child´s age, years | 4.4 | 0.2 | 4.4 | 0.2 | 4.5 | 0.1 | 4.5 | 0.2 | 4.3 | 0.2 | <0.001 |
| Physical activity (parentally reported) |  |  |  |  |  |  |  |  |  |  | <0.001 |
| Sedentary | 683 | 38.1 | 130 | 33.6 | 140 | 35.4 | 216 | 50.3 | 197 | 33.8 |  |
| Active | 1110 | 61.9 | 257 | 66.4 | 255 | 64.6 | 213 | 49.7 | 385 | 66.2 |  |
| TV viewing/day, h | 1.4 | 0.8 | 1.3 | 0.7 | 1.3 | 0.8 | 1.6 | 1.0 | 1.5 | 0.8 | <0.001 |
| Fruit and vegetable intake, 100 g/day | 2.2 | 1.2 | 2.5 | 1.3 | 2.4 | 1.4 | 2.1 | 1.1 | 1.9 | 1.1 | <0.001 |
| Sweetened beverage intake, 100 g/day | 0.8 | 1.1 | 0.6 | 0.7 | 0.4 | 1.0 | 1.1 | 1.4 | 1.1 | 1.1 | <0.001 |
| Daily energy intake, kcal/day | 1582 | 351 | 1644 | 349 | 1474 | 325 | 1633 | 344 | 1576 | 358 | <0.001 |

^a^ *p*-Values from the chi-squared tests for categorical variables and ANOVA tests for continuous variables.
